# Significance of upfront cytoreductive nephrectomy stratified by IMDC risk for metastatic renal cell carcinoma in targeted therapy era – a multi-institutional retrospective study

**DOI:** 10.1007/s10147-021-02091-8

**Published:** 2022-01-01

**Authors:** Renpei Kato, Sei Naito, Kazuyuki Numakura, Shingo Hatakeyama, Tomoyuki Koguchi, Takahiro Kojima, Yoshihide Kawasaki, Shuya Kandori, Sadafumi Kawamura, Yoichi Arai, Akihiro Ito, Hiroyuki Nishiyama, Yoshiyuki Kojima, Chikara Ohyama, Tomonori Habuchi, Norihiko Tsuchiya, Wataru Obara

**Affiliations:** 1 Department of Urology, Iwate Medical University School of Medicine, 2-1-1 Idaidori, Yahaba-cho, Shiwa, 028-3695 Japan; 2 Department of Urology, Yamagata University School of Medicine, 2-2-2 Iida-Nishi, Yamagata, 990-9585 Japan; 3 Department of Urology, Akita University School of Medicine, 1-1-1 Hondo, Akita, 010-8543 Japan; 4 Department of Urology and Advanced Blood Purification Therapy, Hirosaki University Graduate of Medicine, 5 Zaifuchou, Hirosaki, 036-8562 Japan; 5 Department of Urology, Fukushima Medical University School of Medicine, 1 Hikarigaoka, Fukushima, 960-1295 Japan; 6 Department of Urology, University of Tsukuba Graduate School of Medicine, 1-1-1 Tennodai, Tsukuba, 305-8575 Japan; 7 Department of Urology, Tohoku University School of Medicine, 2-1 Seiryo-machi, Aoba-ku, Sendai, 980-8575 Japan; 8 Department of Urology, Miyagi Cancer Center, 47-1, Nodayama, Shiote, Aijima, Natori, 981-1293 Japan

**Keywords:** Upfront cytoreductive nephrectomy, IMDC risk, Japanese, Renal cell carcinoma

## Abstract

**Background:**

This retrospective multicenter study aimed to evaluate the survival benefit of upfront cytoreductive nephrectomy (CN) in metastatic renal cell carcinoma (RCC) patients stratified by International Metastatic RCC Database Consortium (IMDC) risk criteria.

**Methods:**

We reviewed the medical records in the Michinoku Database between 2008 and 2019. Patients who received upfront CN, systemic therapy without CN (no CN) and CN after drug therapy (deferred CN) were analyzed. To exclude selection bias due to patient characteristics, baseline clinical data were adjusted by inverse probability of treatment weighting (IPTW). Overall survival (OS) was compared between upfront CN and non-upfront CN (no CN plus deferred CN). Associations between time-varying covariates including systemic therapies and OS stratified by IMDC risk criteria were analyzed by IPTW-adjusted Cox regression method.

**Results:**

Of 259 patients who fulfilled the selection criteria, 107 were classified in upfront CN and 152 in non-upfront CN group. After IPTW-adjusted analysis, upfront CN showed survival benefit compared to non-upfront CN in patients with IMDC intermediate risk (median OS: 52.5 versus 31.3 months, *p* < 0.01) and in patients with IMDC poor risk (27.2 versus 11.4 months, *p* < 0.01). In IPTW-adjusted Cox regression analysis of time-varying covariates, upfront CN was independently associated with OS benefit in patients with IMDC intermediate risk (hazard ratio 0.52, 95% confidence interval 0.29–0.93, *p* = 0.03) and in patients with IMDC poor risk (0.26, 0.11–0.59, *p* < 0.01).

**Conclusions:**

Upfront CN may confer survival benefit in RCC patients with IMDC intermediate and poor risk.

**Supplementary Information:**

The online version contains supplementary material available at 10.1007/s10147-021-02091-8.

## Introduction

Cytoreductive nephrectomy (CN) for metastatic RCC removes the primary kidney tumor and its potential for bleeding and pain. In addition, CN possibly eliminates the primary tumor as a potential source of immunosuppressive or tumor-promoting growth factors, thus minimizing the risk of future metastatic seeding from primary tumors [1, 2].

Based on two randomized controlled trials showing a survival advantage of nephrectomy plus interferon over interferon alone in early 2000s [3, 4], CN became the standard of care for the management of metastatic RCC nearly 2 decades ago. Since the beginning of the targeted therapy era, the rationale for CN has been based on several retrospective studies demonstrating a survival advantage [5–7]. According to Heng et al. [8], CN is beneficial for synchronous metastatic RCC treated with targeted therapy, even after adjusting for prognostic factors. After the availability of targeted therapy, nephrectomy continued to be used in the clinical setting based on the International Metastatic RCC Database Consortium (IMDC) risk criteria. Recently, the Cancer du Rein Metastatique Nephrectomie et Antiangiogéniques (CARMENA) study, a phase 3 randomized controlled trial, showed no significant prolongation of OS in patients with initial nephrectomy followed by sunitinib compared to patients with sunitinib alone [9].

Although the CARMENA trial is a landmark study in the contemporary management of metastatic RCC, there is concern over possible recruitment bias. This trial supports upfront systemic therapy in patients with high-volume, poor-risk disease and many patients with intermediate-risk disease [9]. Moreover, the generalizability of this trial has not been assessed in a population-based database [10]. Therefore, the benefit and the role of upfront CN remain unclear.

We aimed to evaluate the survival benefit of upfront CN for metastatic RCC stratified by IMDC risk criteria, using inverse probability of treatment weighting (IPTW)-adjusted analyses in real-world clinical setting.

## Materials and methods

### Patients

**Fig. 1:**
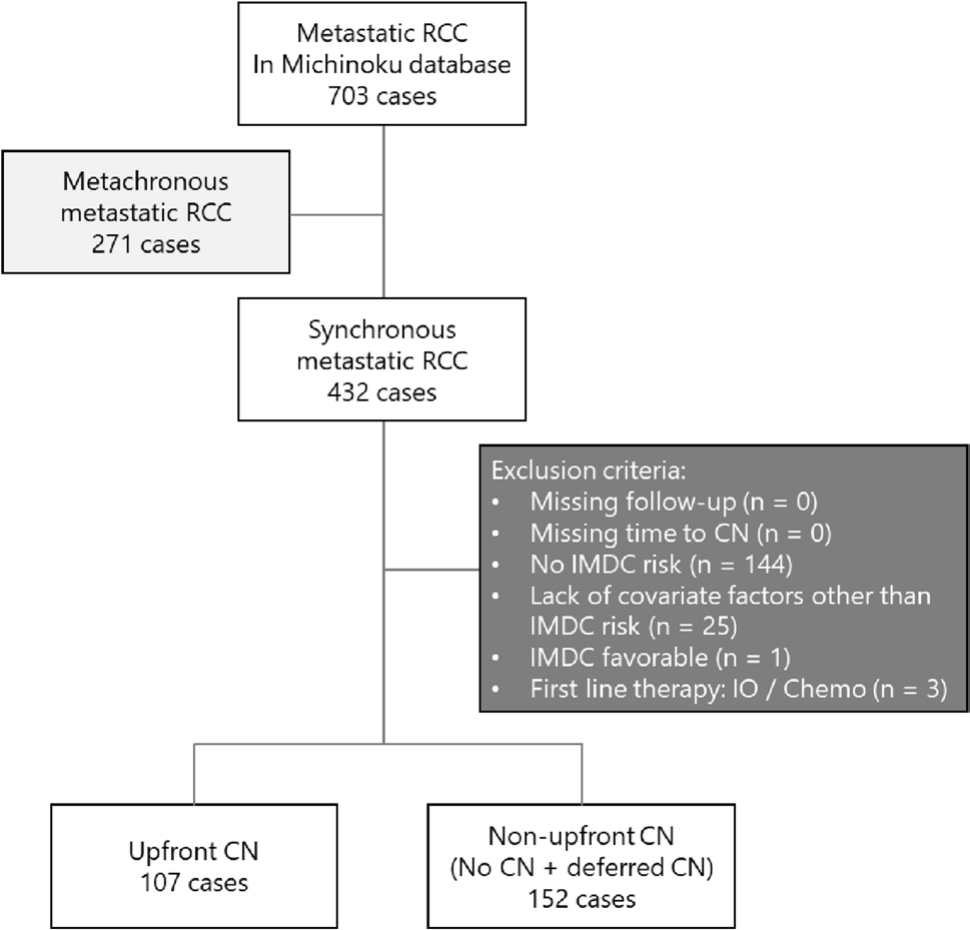
Selection of study cohort from the Michinoku Database and overview of this study. Of 703 patients with renal cell carcinoma (RCC), 432 (61.5%) had synchronous metastases. After excluding 173 patients, 259 patients were studied. Among the included patients, 107 underwent upfront cytoreductive nephrectomy (CN) followed by drug therapy (upfront CN), 125 had only drug therapy or observation without CN (no CN), and 27 received CN after initial drug therapy (deferred CN). To evaluate prognostic significance of upfront CN, we performed comparison between upfront CN group and non-upfront CN group (no CN + deferred CN)

This retrospective study was based on review of medical records in the Michinoku Database from eight institutions between January 2008 and November 2019, as reported previously [11]. The selection process of the study cohort is summarized in Fig. 1. Four hundred and thirty-two consecutive patients who had a confirmed diagnosis of metastatic RCC were enrolled. Exclusion criteria were missing follow-up, missing time to CN, inability to determine IMDC risk status, inability to determine covariate factors other than IMDC risk, IMDC favorable risk, and first-line treatment with immuno-oncology (IO) therapy or chemotherapy. Among these patients, 107 underwent upfront cytoreductive nephrectomy (CN) with or without postoperative drug therapy (upfront CN), 125 had drug therapy or observation only without CN (no CN), and 27 received subsequent CN after drug therapy (deferred CN). Patients with no CN and deferred CN were grouped into non-upfront CN group. Treatments for the subjects were planned according to standard of care in accordance with relevant treatment guidelines. The indication for upfront CN or deferred CN was decided following discussions in the surgical case conference at each institution. Clinical data including medical history, treatment duration, type of systemic therapy, and survival were extracted. This study was conducted in accordance with the ethical principles of the Declaration of Helsinki. The ethical committee of each institutional approved the present study (approval number: MH2019-111). Written informed consent was waived due to the retrospective design.

### Clinical outcome

Types of treatment were identified from the Michinoku database. The sequence of these treatments was determined based on the date of CN and drug therapy initiation documented in the database. The primary outcome measure was overall survival (OS) in upfront CN and non-upfront CN groups. Subgroup analyses stratified by IMDC risk criteria were performed.

### Comparison between patients with longer OS and those with shorter OS in upfront CN group

To evaluate the clinical features of patients who potentially benefit from upfront CN, we compared patients with longer OS (equal to or longer than median OS) and those with shorter OS (shorter than median OS) in upfront CN group with IMDC intermediate risk and poor risk. The clinical factors analyzed were those used for calculating IMDC risk score [Eastern Cooperative Oncology Group (ECOG) performance status (PS), hemoglobin (Hb), corrected calcium, neutrophil count, and platelet count], other known prognostic factors of CN [albumin (Alb), C-reactive protein (CRP), and neutrophil-to-lymphocyte ratio (NLR)] [8, 12–14] and number of metastases (lung, bone, liver and brain) that were not included in the propensity score model.

### Statistical analysis

Fisher’s exact test and t-test were used to compare patient characteristics and types of systemic therapy. Spearman's rank correlation coefficient was used to compare the time of initiation of the first systemic therapy.

To adjust for differences in baseline characteristics that may have influenced the initial treatment selection, we performed IPTW based on the propensity to receive upfront CN versus non-upfront CN, which was estimated by logistic regression. Supplementary Table 1 shows the variables included. These variables were selected based on their association with age-sex-specific mortality and cancer-specific mortality [15–18]. The covariate balance was assessed by standardized differences. A standardized difference < 0.1 was considered a negligible difference in the mean or prevalence of a covariate between two groups [19].

OS was calculated from the date of diagnosis of metastatic RCC to the date of all-cause death or date of the last follow-up. Survival curves were constructed using the Kaplan–Meier method and analyzed by the log-rank test. In addition, an IPTW-adjusted Cox proportional hazard regression analysis was performed to estimate the prognostic significance of time-varying covariates including systemic treatments [20]. Correlation between outcome and the variables is expressed as hazard ratio (HR) and 95% confidence interval (CI). In upfront CN group with IMDC intermediate and poor risk, clinical parameters were compared between patients with longer OS and those with shorter OS by Fisher’s exact test and t-test. All statistical analyses were conducted using JMP 14.3.0 (SAS Institute, Cary, NC).

## Results

### Patients’ characteristics and outcome in all subjects

Two hundred and fifty-nine patients fulfilled the selection criteria in this study (Fig. 1). To evaluate the clinical significance of upfront CN, we divided all patients into an upfront CN group (*n* = 107) and a non-upfront CN group (no CN and deferred CN; *n* = 152). Median follow-up period was 21.3 months (range, 0.2‒147.6).

Patient characteristics of the two groups are summarized in Table 1. Patients who underwent upfront CN were younger, with lower proportions of IMDC poor risk, ECOG PS 1 or higher, clinical T3 and T4 primary tumors, cN1 disease and liver metastasis. The proportions of patients treated with cytokine therapy, subsequent nivolumab monotherapy and metastasectomy were higher in upfront CN group than in non-upfront CN group. Drugs used as first-line therapy are summarized in Supplementary Table 2. The most frequently used targeted therapy was sunitinib, and the most frequently used cytokine therapy was interferon monotherapy. After IPTW adjustment, the baseline cohort characteristics were balanced except for the proportion of patients with brain metastasis (Table 2, Supplementary Fig. 1A and B).

**Table 1:** Patient characteristics of study groups

Characteristics	Upfront CN *n* = 107	Non-upfront CN *n* = 152	*p* value
Age, mean ± SD (years)	64.2 ± 1.0	67.0 ± 0.9	0.03
Sex (men), *n* (%)	77 (72.0)	110 (72.4)	1.00
IMDC intermediate / poor risk, n	70 / 37	57 / 95	< 0.01
ECOG PS ≥ 1, *n* (%)	30 (28.0)	74 (48.7)	< 0.01
T stage cT3 / 4, *n* (%)	63 (58.9)	116 (76.3)	< 0.01
N stage cN1 / 2, *n* (%)	31 (29.0)	71 (46.7)	< 0.01
M stage cM1, *n* (%)	99 (92.5)	141 (92.8)	1.00
Metastatic sites, *n* (%)			
Lung	72 (67.3)	100 (65.8)	0.89
Bone	33 (30.8)	49 (32.2)	0.89
Liver	9 (8.4)	27 (17.8)	0.04
Brain	8 (7.5)	13 (8.6)	0.82
Lymph node	35 (32.7)	55 (36.2)	0.60
First line drug therapy,			
Cytokine /targeted therapy / none, *n*	20 / 81 / 6	6 / 141 / 5	< 0.01
Subsequent NIVO monotherapy	36 (33.6)	20 (13.2)	< 0.01
Metastasectomy, *n* (%)	23 (21.5)	11 (7.2)	< 0.01
RT for bone metastases, *n* (%)	26 (24.3)	24 (15.8)	0.11
RT for brain metastases, *n* (%)	12 (11.2)	10 (6.6)	0.26

*CN* Cytoreductive nephrectomy, *IMDC* the International Metastatic RCC Database Consortium, *ECOG PS* ECOG performance status, *NIVO* nivolumab, *RT* radiation therapy

**Table 2:** Baseline characteristics of all patients divided into upfront CN group and non-upfront CN group: unadjusted and IPTW-adjusted cohorts

Characteristics	Unadjusted cohort			IPTW-adjusted cohort		
	Upfront CN *n* = 107	Non-upfront CN *n* = 152	Std diff	Upfront CN *n* = 267	Non-upfront CN *n* = 254	Std diff
Age, mean ± SD (year)	64.2 ± 1.0	67.0 ± 0.9	0.27	65.6 ± 0.6	65.9 ± 0.7	0.03
Sex (men), *n* (%)	77 (72.0)	110 (72.4)	0.01	191 (71.3)	183 (72.2)	0.01
IMDC poor risk, *n* (%)	37 (34.6)	95 (62.5)	0.56	144 (53.8)	133 (52.4)	0.03
ECOG PS ≥ 1, *n* (%)	30 (28.0)	74 (48.7)	0.53	113 (42.4)	105 (41.2)	0.03
T stage cT3/4, *n* (%)	63 (58.9)	116 (76.3)	0.26	191 (71.3)	180 (71.1)	0.00
N stage cN1/2, *n* (%)	31 (29.0)	71 (46.7)	0.46	120 (44.7)	106 (41.8)	0.07
M stage cM1, *n* (%)	99 (92.5)	141 (92.8)	0.00	248 (92.9)	236 (93.0)	0.00
Metastatic sites, *n* (%)						
Lung	72 (67.3)	100 (65.8)	0.02	182 (68.0)	168 (66.1)	0.03
Bone	33 (30.8)	49 (32.2)	0.05	78 (29.2)	79 (31.2)	0.07
Liver	9 (8.4)	27 (17.8)	0.70	39 (14.5)	36 (14.4)	0.01
Brain	8 (7.5)	13 (8.6)	0.15	16 (6.0)	18 (7.1)	0.18
Lymph node	35 (32.7)	55 (36.2)	0.10	99 (37.1)	89 (35.1)	0.06

*IPTW* inverse probability of treatment weighting, *CN* cytoreductive nephrectomy, *IMDC* the International Metastatic RCC Database Consortium, *ECOG PS* ECOG performance status, *Std diff* standardized difference

**Fig. 2:**
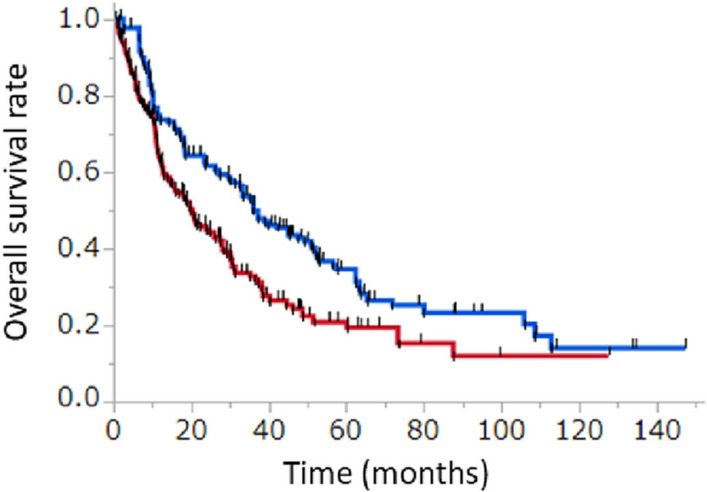
IPTW-adjusted overall survival (OS) in all subjects. Red curve represents non-upfront CN group, and blue curve represents upfront CN group. IPTW-adjusted OS is significantly longer in upfront CN group than in non-upfront CN group [36.1 (32.6–45.1) months versus 20.4 (15.6 – 26.0) months; *p* < 0.01]. CN; cytoreductive nephrectomy, IPTW; inverse probability of treatment weighting

Figure 2 shows IPTW-adjusted OS of upfront CN and non-upfront CN groups. The upfront CN group showed significantly longer OS compared to non-upfront CN group after IPTW adjustment of baseline characteristics [median OS: 36.1 (95% CI 32.6‒45.1) months versus 20.4 (95% CI 15.6‒26.0) months, *p* < 0.01] (Fig. 2). Analysis using unadjusted baseline characteristics also yielded significantly longer OS in upfronted CN group compared to non-upfronted CN group (Supplementary Fig. 2). Year of initiation of the first therapy was similar between the two groups (Supplementary Fig. 3).

### Subgroup analyses stratified by IMDC risk criteria

**Fig. 3:**
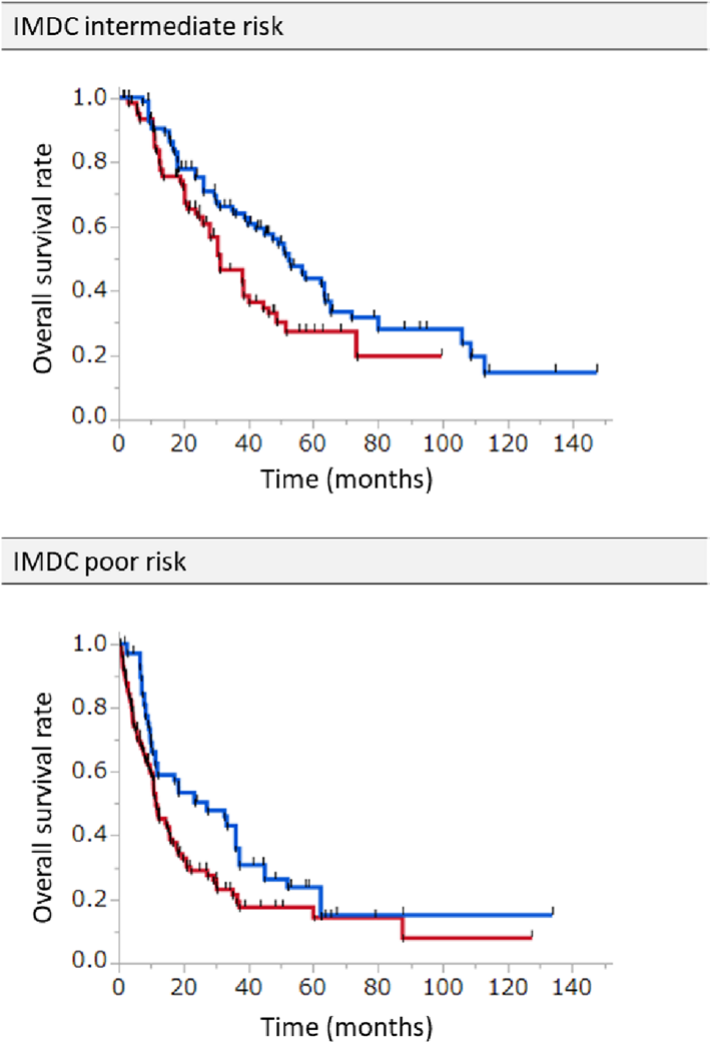
IPTW-adjusted overall survival (OS) in patients with IMDC intermediate or poor risk. Red curve represents non-upfront CN group, and blue curve represents upfront CN group. IMDC intermediate risk subgroup: IPTW-adjusted OS is significantly longer in the upfront CN group than in the non-upfront CN group [52.5 (42.1–63.4) months versus 31.3 (28.1–38.5) months; *p* < 0.01]. IMDC poor risk group: IPTW-adjusted OS is significantly longer in the upfront CN group than in the non-upfront CN group [27.2 (11.9 – 36.1) months versus 11.4 (10.4 – 15.7) months; *p* < 0.01]. *CN* cytoreductive nephrectomy, *IPTW* inverse probability of treatment weighting

Clinical characteristics of patients with IMDC intermediate risk are summarized in Table 3. In this subgroup, patients who underwent upfront CN had less IMDC risk factors, and lower proportions of clinical T3 and T4 primary tumors, cN1 disease, and liver metastasis. After IPTW adjustment, the baseline cohort characteristics were balanced except for the proportions of patients with ECOG PS 1 or higher and brain metastasis (Table 3). Upfront CN was associated with significantly longer OS compared to non-upfront CN after IPTW adjustment of baseline characteristics [median OS: 52.5 (95% CI 42.1‒63.4) months versus 31.3 (95% CI 28.1‒38.5) months, *p* < 0.01] (Fig. 3).

**Table 3:** Baseline characteristics of patients with IMDC intermediate risk divided into upfront CN group and non-upfront CN group: unadjusted and IPTW-adjusted cohorts

Characteristics	Unadjusted cohort			IPTW-adjusted cohort			
	Upfront CN *n* = 70	Non-upfront CN *n* = 57	*p* value	Std diff	Upfront CN *n* = 125	Non-upfront CN *n* = 125	Std diff
Age, mean ± SD (year)	63.6 ± 1.3	67.3 ± 1.5	0.06	0.34	64.3 ± 1.0	64.9 ± 1.1	0.05
Sex (men), *n* (%)	48 (68.6)	42 (73.7)	0.56	0.07	87 (69.8)	86 (68.8)	0.01
Number of IMDC risk:							
2 risks, *n* (%)	38 (54.3)	42 (73.7)	0.03	0.30	77 (62.0)	78 (62.7)	0.01
ECOG PS ≥ 1, *n* (%)	10 (14.3)	8 (14.0)	1.00	0.02	18 (14.3)	20 (16.3)	0.13
T stage cT3b/3c/4, *n* (%)	37 (52.9)	43 (75.4)	0.01	0.35	80 (64.0)	87 (69.9)	0.09
N stage cN1/2, n (%)	13 (18.6)	25 (43.9)	< 0.01	0.76	37 (29.9)	40 (31.9)	0.07
M stage cM1, *n* (%)	65 (92.9)	51 (89.5)	0.54	0.04	116 (93.1)	116 (92.9)	0.00
Metastatic sites, *n* (%)							
Lung	46 (65.7)	38 (66.7)	1.00	0.02	84 (67.7)	80 (64.3)	0.05
Bone	23 (32.9)	12 (21.1)	0.16	0.43	36 (28.9)	38 (30.3)	0.05
Liver	4 (5.7)	10 (17.5)	0.04	0.94	13 (10.2)	13 (10.7)	0.05
Brain	5 (7.1)	1 (1.8)	0.22	1.12	6 (4.6)	3 (2.1)	0.81
Lymph node	17 (24.3)	19 (33.3)	0.32	0.31	33 (26.1)	36 (28.9)	0.10

*IPTW* inverse probability of treatment weighting, *CN* cytoreductive nephrectomy, *IMDC* the International Metastatic RCC Database Consortium, *ECOG PS* ECOG performance status, *Std diff* standardized difference

Clinical characteristics of patients with IMDC poor risk are summarized in Table 4. There were no significant differences in patient characteristics between the two groups. After IPTW adjustment, the baseline cohort characteristics were balanced except for the proportions of patients with liver and brain metastases (Table 4). Upfront CN was associated with significantly longer OS compared to non-upfront CN after IPTW adjustment of baseline characteristics [median OS: 27.2 (95% CI 11.9‒36.1) months versus 11.4 (95% CI 10.4‒15.7) months, *p* < 0.01] (Fig. 3).

**Table 4:** Baseline characteristics of patients with IMDC poor risk divided into upfront CN group and non-upfront CN group: unadjusted and IPTW-adjusted cohorts

Characteristics	Unadjusted cohort				IPTW-adjusted cohort		
	Upfront CN *n* = 37	Non-upfront CN *n* = 95	*p* value	Std diff	Upfront CN *n* = 132	Non-upfront CN *n* = 131	Std diff
Age, mean ± SD (year)	65.3 ± 1.3	66.9 ± 1.0	0.36	0.18	66.4 ± 0.7	66.4 ± 0.9	0.00
Sex (men), *n* (%)	29 (78.4)	68 (71.6)	0.51	0.09	94 (71.2)	96 (73.2)	0.03
Number of IMDC risk: ≥ 4 risks, *n* (%)	16 (43.2)	46 (48.4)	0.70	0.11	59 (45.0)	62 (47.2)	0.05
ECOG PS ≥ 1, *n* (%)	20 (54.1)	66 (69.5)	0.11	0.25	90 (68.3)	87 (66.3)	0.03
T stage cT3b/3c/4, *n* (%)	26 (70.3)	73 (76.8)	0.50	0.09	100 (75.8)	99 (75.5)	0.00
N stage cN1/2, *n* (%)	18 (48.7)	46 (48.4)	1.00	0.01	69 (52.0)	65 (49.4)	0.05
M stage cM1, *n* (%)	34 (91.9)	90 (94.7)	0.69	0.03	125 (94.6)	124 (94.3)	0.00
Metastatic sites, *n* (%)							
Lung	26 (70.3)	62 (65.3)	0.68	0.07	94 (71.5)	88 (67.1)	0.06
Bone	10 (27.0)	37 (39.0)	0.23	0.36	46 (34.7)	47 (36.1)	0.04
Liver	5 (13.5)	17 (17.9)	0.61	0.29	19 (14.2)	22 (16.7)	0.17
Brain	3 (8.1)	12 (12.6)	0.56	0.45	12 (8.8)	15 (11.3)	0.26
Lymph node	18 (48.7)	36 (37.9)	0.32	0.25	56 (42.0)	54 (41.4)	0.01

*IPTW* inverse probability of treatment weighting, *CN* cytoreductive nephrectomy, *IMDC* the International Metastatic RCC Database Consortium, *ECOG PS* ECOG performance status, *Std diff *standardized difference

In both IMDC intermediate and poor risk patients, analysis with unadjusted baseline characteristics also showed significantly longer OS in the upfront CN group than in the non-upfront CN group (Supplementary Fig. 4).

### Association of upfront CN and other systemic therapies with survival

The upfront CN and non-upfront CN groups differed significantly not only in baseline characteristics but also in the use of some systemic therapies (Table 1, Supplementary Table 3). In the subgroup with IMDC intermediate risk, the proportions of patients treated with cytokine therapy, subsequent nivolumab monotherapy, metastasectomy and radiotherapy for brain metastasis were higher in upfront CN group than in non-upfront CN. In the subgroup with IMDC poor risk, the proportion of patients treated with cytokine therapy and subsequent nivolumab monotherapy was higher in upfront CN than in non-upfront CN. Given these differences in systemic treatment, we performed an IPTW-adjusted Cox regression analysis to estimate the prognostic significance of time-varying covariates including systemic treatments.

In the subgroup with IMDC intermediate risk, univariate and multivariate analyses of IPTW-adjusted cohort identified upfront CN versus no CN as an independent factor associated with prolonged OS, and older age and clinical T3 and T4 primary tumors as independent factors associated with shortened OS (Supplementary Table 4).

In the subgroup with IMDC poor risk, univariate and multivariate analyses of IPTW-adjusted cohort identified upfront CN versus no CN and metastasectomy as independently associated with prolonged OS (Table 5).

**Table 5:** IPTW-adjusted univariate and multivariate Cox regression analyses for systemic therapies predicting overall survival in IMDC poor risk group

Covariates		Univariate analysis			Multivariate analysis		
		HR	95% CI	*p* value	HR	95% CI	*p* value
Age	≥ 75 vs 75 >	1.08	0.57–2.03	0.82	1.13	0.40–3.19	0.82
Sex	Men vs Female	0.66	0.42–1.05	0.08	0.50	0.20–1.24	0.14
Number of IMDC risk:	≥ 4 risks vs 3 risks	0.69	0.40–1.21	0.20	0.35	0.13–0.95	0.04
ECOG PS	≥ 1 vs 0	1.25	0.78–1.99	0.36	1.28	0.60–2.72	0.52
T stage	≥ cT3 vs cT2 ≥	1.12	0.67–1.87	0.66	0.67	0.31–1.43	0.30
N stage	cN1/2 vs cN0	1.65	1.04–2.61	0.03	0.92	0.38–2.21	0.85
M stage	cM1 vs cM0	0.58	0.17–1.95	0.38	0.24	0.05–1.17	0.08
Metastatic sites							
Lung	Yes vs No	1.26	0.78–2.04	0.34	1.21	0.55–2.69	0.63
Bone	Yes vs No	1.42	0.78–2.61	0.25	4.98	1.77–14.0	< 0.01
Liver	Yes vs No	0.77	0.38–1.56	0.47	0.42	0.13–1.44	0.17
Brain	Yes vs No	1.92	0.86–4.29	0.11	4.32	1.14–16.4	0.03
Lymph node	Yes vs No	0.93	0.57–1.51	0.76	3.02	1.24–7.36	0.02
CN							
	U-CN vs No-CN	0.45	0.25–0.81	< 0.01	0.22	0.09–0.50	< 0.01
	U-CN vs D-CN	1.01	0.54–1.88	0.97	0.41	0.14–1.23	0.11
	D-CN vs No-CN	0.45	0.21–0.95	0.04	0.53	0.17 –1.66	0.27
First line drug therapy	Cy vs TT	0.73	0.23–2.36	0.60	3.03	0.61–15.0	0.17
Subsequent NIVO monotherapy	Yes vs No	0.95	0.56–1.59	0.83	1.80	0.74–4.36	0.20
Metastasectomy	Yes vs No	0.37	0.16–0.87	0.02	0.11	0.02–0.54	< 0.01
RT for bone metastases	Yes vs No	0.42	0.16–1.10	0.08	0.20	0.06–0.68	< 0.01
RT for brain metastases	Yes vs No	1.00	0.29–3.49	0.99	0.64	0.07–5.68	0.69

*U-CN* upfront cytoreductive nephrectomy, *D-CN* deferred cytoreductive nephrectomy, *No-CN* no cytoreductive nephrectomy, *Cy* cytokine therapy, *TT* targeted therapy, *NIVO* nivolumab, *RT* radiation therapy, *N.D.* not detected

### Clinical features of patients with longer OS versus shorter OS in upfront CN group

To evaluate the clinical features of IMDC intermediate and poor risk patients who potentially benefit from upfront CN, we analyzed the IMDC risk factors, other known prognostic factors of CN and number of metastatic sites not included in propensity score model (Table 6). In IPTW-adjusted cohort with IMDC intermediate risk, mean number of lung metastases was significantly smaller in patients with longer OS than in those with shorter OS (1.7 ± 0.3 versus 3.2 ± 0.3, *p* < 0.01).

**Table 6:** Comparison of clinical features between patients with longer overall survival (OS) and those with shorter OS in the upfront cytoreductive nephrectomy group

	Unadjusted cohort			IPTW-adjusted cohort		
	Longer OS	Shorter OS	*p* value	Longer OS	Shorter OS	*p* value
IMDC intermediate risk						
ECOG PS ≥ 2, n (%)	2 (5.7)	2 (5.7)	1.00	3 (5.1)	4 (6.2)	0.77
Hb, mean ± SD (g/dL)	13.9 ± 0.3	13.1 ± 0.5	0.20	13.8 ± 0.2	13.0 ± 0.4	0.08
Corrected calcium, mean ± SD (mg/dL)	9.1 ± 0.1	9.1 ± 0.1	1.00	9.1 ± 0.1	9.1 ± 0.1	0.86
Neutrophil count, mean ± SD (× 10^3^/µL)	4.3 ± 0.2	4.3 ± 0.3	0.96	4.2 ± 0.1	4.3 ± 0.2	0.73
Platelet count, mean ± SD (× 10^4^/µL)	24.1 ± 0.1	24.3 ± 0.1	0.91	23.8 ± 0.1	24.4 ± 0.1	0.67
Alb, mean ± SD (g/dL)	4.1 ± 0.1	4.0 ± 0.1	0.42	4.1 ± 0.1	3.9 ± 0.1	0.16
CRP, mean ± SD (mg/dL)	1.5 ± 0.4	2.3 ± 0.7	0.35	1.4 ± 0.3	2.4 ± 0.5	0.08
NLR, mean ± SD	2.8 ± 0.2	2.9 ± 0.2	0.85	2.8 ± 0.1	2.9 ± 0.2	0.50
Number of metastases, mean ± SD						
Lung	1.9 ± 0.4	3.0 ± 0.4	0.06	1.7 ± 0.3	3.2 ± 0.3	< 0.01
Bone	0.7 ± 0.2	0.7 ± 0.3	0.92	0.7 ± 0.1	0.7 ± 0.2	0.93
Liver	0.2 ± 0.2	0.2 ± 0.2	0.79	0.2 ± 0.1	0.3 ± 0.1	0.96
Brain	0.1 ± 0.1	0.2 ± 0.1	0.31	0.1 ± 0.0	0.1 ± 0.1	0.30
IMDC poor risk						
ECOG PS ≥ 2, n (%)	3 (16.7)	2 (10.5)	0.66	15 (20.5)	7 (9.3)	0.16
Hb, mean ± SD (g/dL)	10.6 ± 0.4	11.1 ± 0.4	0.35	10.6 ± 0.2	11.3 ± 0.2	< 0.01
Corrected calcium, mean ± SD (mg/dL)	9.7 ± 0.3	9.5 ± 0.2	0.55	9.7 ± 0.1	9.7 ± 0.1	0.89
Neutrophil count, mean ± SD (× 10^3^/µL)	4.6 ± 0.4	5.6 ± 0.5	0.16	4.7 ± 0.2	5.4 ± 0.3	0.08
Platelet count, mean ± SD (× 10^4^/µL)	37.0 ± 0.3	33.6 ± 0.2	0.40	35.6 ± 0.1	33.2 ± 0.1	0.22
Alb, mean ± SD (g/dL)	3.0 ± 0.2	3.3 ± 0.1	0.15	3.0 ± 0.1	3.4 ± 0.1	< 0.01
CRP, mean ± SD (mg/dL)	7.9 ± 1.7	6.4 ± 1.3	0.50	7.7 ± 0.8	5.4 ± 0.6	0.02
NLR, mean ± SD	2.8 ± 0.2	2.9 ± 0.2	0.85	2.8 ± 0.1	2.9 ± 0.2	0.50
Number of metastases, mean ± SD						
Lung	2.4 ± 0.6	2.3 ± 0.5	0.90	2.3 ± 0.3	2.2 ± 0.2	0.94
Bone	0.1 ± 0.1	1.1 ± 0.4	0.02	0.1 ± 0.0	1.2 ± 0.2	< 0.01
Liver	0.4 ± 0.3	0.1 ± 0.1	0.33	0.5 ± 0.1	0.2 ± 0.0	0.12
Brain	0.1 ± 0.1	0.1 ± 0.1	0.50	0.1 ± 0.0	0.0 ± 0.0	0.42

Longer OS; ≥ median OS, Shorter OS; < median OS, *IPTW* inverse probability of treatment weighting, *IMDC* the International Metastatic RCC Database Consortium, *CN* cytoreductive nephrectomy, *ECOG PS* ECOG performance status, *Hb* hemoglobin, *Alb* albumin, *CRP* C-reactive protein, *NLR* neutrophil-to-lymphocyte ratio, *SD* standard deviation

In IPTW-adjusted cohort with IMDC poor risk, mean Hb level was significantly lower (10.6 ± 0.2 versus 11.3 ± 0.2 mg/dL, *p* < 0.01) and mean albumin-corrected calcium level was also significantly lower (3.0 ± 0.1 versus 3.4 ± 0.1 mg/dL, *p* < 0.01) in patients with longer OS than in those with shorter OS. Mean CRP was significantly higher for longer OS than for shorter OS (7.7 ± 0.80 versus 5.4 ± 0.6 mg/dL, *p* = 0.02). Mean number of bone metastases was significantly smaller for longer OS than for shorter OS (0.1 ± 0.1 versus 1.2 ± 0.2, *p* < 0.01).

## Discussion

Our study using IPTW analysis demonstrated that upfront CN confers survival benefit in patients with IMDC intermediate and poor risk. For patients diagnosed with metastatic RCC, a multidisciplinary conference to decide treatment options should be conducted, including discussions on whether CN is likely to provide survival benefit.

The CARMENA trial reported no significant prolongation of OS in patients who received initial nephrectomy followed by sunitinib compared to patients with sunitinib alone. Although the CARMENA trial is an important and commendable effort to evaluate the impact of CN in patients with metastatic RCC, it should be evaluated within the context of its limitations [1]. Recently, our group reported that CN conferred survival benefit in metastatic RCC patients treated with targeted therapy using IPTW analysis. In that study, OS in patients treated with upfront CN was significantly longer than in those without CN. Patients treated with deferred CN also had prolonged OS compared with those without CN [11]. In the clinical setting, deferred CN is often selected for patients who has achieved good response to first-line drug therapy. The no-CN group included patients who planned deferred CN at the start of first-line therapy, but continued drug therapy because of insufficient response. In the present study, we aimed to verify whether CN or drug therapy should be selected as the first-line therapy by performing analyses comparing patients with upfront CN and those with no CN + deferred CN. In this study, the upfront CN group had more patients treated with cytokine therapy than the no CN group. Naito, et al. [21] showed that cytokine therapy conferred survival benefit in Japanese RCC patients. Based on this evidence, cytokine therapy has been used as standard of care for eligible patients in the era of targeted therapy in Japan. Hence, inclusion of patients on targeted therapy and those on cytokine therapy is important to verify the benefit of upfront CN with respect to IMDC risk in the real-world clinical setting in Japan.

The present study demonstrated that upfront CN confers survival benefit in patients with IMDC intermediate or poor risk, even after adjusting for baseline patient characteristics. IPTW-adjusted Cox regression analyses in patients with IMDC intermediate risk showed that ECOG PS 1 or higher and the presence of brain metastasis were not associated with worse OS. Furthermore, the presence of brain or liver metastasis was not associated with worse OS in IPTW-adjusted Cox regression analyses in patients with IMDC poor risk. However, the OS curve of patients with IMDC poor risk decreased steeply within 12 months (Fig. 3D). We consider that patients with IMDC poor risk who are expected to survive less than 12 months may have marginal benefit from upfront CN.

The present study showed differences in the proportions of patients using systemic therapies other than CN between upfront CN and non-upfront CN groups, after adjusting patient characteristics. In RCC patients, targeted therapy prolonged survival compared with cytokine therapy [22], and local therapy by metastasectomy and radiation therapy for bone or brain metastasis improved clinical outcome [23–28]. To verify whether other systemic therapies contributed to the prolonged OS observed with upfront CN, we performed multivariate analysis including upfront CN and other systemic therapies in IPTW-adjusted Cox proportional hazard regression models. In the subgroup with IMDC intermediate risk, upfront CN versus no CN was identified as an independent factor predicting OS prolongation, while other systemic therapies were not associated with OS. Therefore, the option of upfront CN should be considered at the beginning of treatment in patients with IMDC intermediate risk. In patients with IMDC poor risk, upfront CN versus no CN and metastasectomy were independently associated with OS benefit. Therefore, upfront CN may also be indicated in metastatic RCC patients with IMDC poor risk.

To evaluate the clinical features of patients who potentially benefit from upfront CN, we analyzed factors including IMDC risk factors and other known prognostic factors of CN [8, 12–14]. In our analysis of upfront CN group with IMDC intermediate risk, patients with longer OS had less lung metastases. In patients with IMDC intermediate risk who had small volume of lung metastases, targeted therapy subsequent to upfront CN may bring survival benefit. On the other hand, in our analysis of upfront CN group with IMDC poor risk, IPTW-adjusted cohort with longer OS showed significantly lower Hb, lower albumin level, and higher CRP level compared to those with shorter OS. However, all these factors did not differ significantly between the longer and shorter OS groups in IPTW-unadjusted cohort. These differences between IPTW-unadjusted cohort and IPTW-adjusted cohort may be due to overweighted effects of minority group by IPTW analysis. In both IPTW-unadjusted and -adjusted analyses, patients with shorter OS had more bone metastases than patients with longer OS. As reported previously, the presence of bone metastases is an unfavorable prognostic factor [29]. Indications for upfront CN in patients with IMDC poor risk have not yet been established. The present analysis suggests that IMDC poor risk patients who have multiple bone metastases may not benefit from upfront CN in terms of overall survival. Refining the risk stratification model is crucial for determining which patient may benefit from upfront CN. IO combination therapy prolonged progression-free survival and OS in patients with bone metastases compared with sunitinib [30]. Further study is required to evaluate the survival benefit of upfront CN in IMDC poor risk patients with bone metastases in the IO therapy era.

Recent studies have demonstrated that institutional experience is associated with improved outcomes [31, 32]. In our study, most participating institutions are academic centers. This may be one of the factors that contributed to better survival with upfront CN.

Our study had several limitations. First, it was a retrospective study with a small sample size and potential selection bias. Even though we performed IPTW-adjusted analyses, this could not control several unmeasured confounders. Second, the indication for upfront CN was not uniform among the participating institutions. Third, we excluded patients treated with first-line IO therapy, because only one patient had IO therapy. Finally, the IPTW method has overweighted effects of minority group, resulting in an adjusted cohort that differs to a great extent from the median patient characteristics. Further study is required to evaluate the survival benefit of upfront CN followed by IO therapy compared to IO therapy alone. Nevertheless, to the best of our knowledge, this is the first report on the prognostic significance of upfront CN stratified by IMDC risk criteria, using IPTW-adjusted analysis. Nowadays, IO combination therapies have become the standard of care for metastatic RCC patients. These novel agents are expected to prolong survival after upfront CN.

In conclusion, our study found that upfront CN conferred survival benefit in Japanese patients with IMDC intermediate and poor risk. Careful patient selection is warranted to determine if a patient will benefit from CN.

## Supplementary Information

Below is the link to the electronic supplementary material.

Supplementary file1 (DOCX 41 KB)

Supplementary file2 (DOCX 628 KB)
