# Correction to: Significance of upfront cytoreductive nephrectomy stratified by IMDC risk for metastatic renal cell carcinoma in targeted therapy era – a multi‑institutional retrospective study

**DOI:** 10.1007/s10147-023-02331-z

**Published:** 2023-04-03

**Authors:** Renpei Kato, Sei Naito, Kazuyuki Numakura, Shingo Hatakeyama, Tomoyuki Koguchi, Takahiro Kojima, Yoshihide Kawasaki, Shuya Kandori, Sadafumi Kawamura, Yoichi Arai, Akihiro Ito, Hiroyuki Nishiyama, Yoshiyuki Kojima, Chikara Ohyama, Tomonori Habuchi, Norihiko Tsuchiya, Wataru Obara

**Affiliations:** 1 Department of Urology, Iwate Medical University School of Medicine, 2-1-1 Idaidori, Yahaba-cho, Shiwa, 028-3695 Japan; 2 Department of Urology, Yamagata University School of Medicine, 2-2-2 Iida-Nishi, Yamagata, 990-9585 Japan; 3 Department of Urology, Akita University School of Medicine, 1-1-1 Hondo, Akita, 010-8543 Japan; 4 Department of Urology and Advanced Blood Purification Therapy, Hirosaki University Graduate of Medicine, 5 Zaifuchou, Hirosaki, 036-8562 Japan; 5 Department of Urology, Fukushima Medical University School of Medicine, 1 Hikarigaoka, Fukushima, 960-1295 Japan; 6 Department of Urology, University of Tsukuba Graduate School of Medicine, 1-1-1 Tennodai, Tsukuba, 305-8575 Japan; 7 Department of Urology, Tohoku University School of Medicine, 2-1 Seiryo-machi, Aoba-ku, Sendai, 980-8575 Japan; 8 Department of Urology, Miyagi Cancer Center, 47-1, Nodayama, Shiote, Aijima, Natori, 981-1293 Japan

**Correction to: International Journal of Clinical Oncology (2022) 27:563–573** 10.1007/s10147-021-02091-8

In the original publication, in Table 6, the p-values of Number of metastases, mean ± SD under IMDC intermediate risk were published incorrectly. The corrected version of Table 6 is given in this correction.

**Table 6:** Comparison of clinical features between patients with longer overall survival (OS) and those with shorter OS in the upfront cytoreductive nephrectomy group

	Unadjusted cohort			IPTW-adjusted cohort		
	Longer OS	Shorter OS	*p* value	Longer OS	Shorter OS	*p* value
IMDC intermediate risk						
ECOG PS ≥ 2, n (%)	2 (5.7)	2 (5.7)	1.00	3 (5.1)	4 (6.2)	0.77
Hb, mean ± SD (g/dL)	13.9 ± 0.3	13.1 ± 0.5	0.20	13.8 ± 0.2	13.0 ± 0.4	0.08
Corrected calcium, mean ± SD (mg/dL)	9.1 ± 0.1	9.1 ± 0.1	1.00	9.1 ± 0.1	9.1 ± 0.1	0.86
Neutrophil count, mean ± SD (× 10^3^/µL)	4.3 ± 0.2	4.3 ± 0.3	0.96	4.2 ± 0.1	4.3 ± 0.2	0.73
Platelet count, mean ± SD (× 10^4^/µL)	24.1 ± 0.1	24.3 ± 0.1	0.91	23.8 ± 0.1	24.4 ± 0.1	0.67
Alb, mean ± SD (g/dL)	4.1 ± 0.1	4.0 ± 0.1	0.42	4.1 ± 0.1	3.9 ± 0.1	0.16
CRP, mean ± SD (mg/dL)	1.5 ± 0.4	2.3 ± 0.7	0.35	1.4 ± 0.3	2.4 ± 0.5	0.08
NLR, mean ± SD	2.8 ± 0.2	2.9 ± 0.2	0.85	2.8 ± 0.1	2.9 ± 0.2	0.50
Number of metastases, mean ± SD						
Lung	1.9 ± 0.4	3.0 ± 0.4	0.06	1.7 ± 0.3	3.2 ± 0.3	< 0.01
Bone	0.7 ± 0.2	0.7 ± 0.3	0.92	0.7 ± 0.1	0.7 ± 0.2	0.93
Liver	0.2 ± 0.2	0.2 ± 0.2	0.79	0.2 ± 0.1	0.3 ± 0.1	0.96
Brain	0.1 ± 0.1	0.2 ± 0.1	0.31	0.1 ± 0.0	0.1 ± 0.1	0.30
IMDC poor risk						
ECOG PS ≥ 2, n (%)	3 (16.7)	2 (10.5)	0.66	15 (20.5)	7 (9.3)	0.16
Hb, mean ± SD (g/dL)	10.6 ± 0.4	11.1 ± 0.4	0.35	10.6 ± 0.2	11.3 ± 0.2	< 0.01
Corrected calcium, mean ± SD (mg/dL)	9.7 ± 0.3	9.5 ± 0.2	0.55	9.7 ± 0.1	9.7 ± 0.1	0.89
Neutrophil count, mean ± SD (× 10^3^/µL)	4.6 ± 0.4	5.6 ± 0.5	0.16	4.7 ± 0.2	5.4 ± 0.3	0.08
Platelet count, mean ± SD (× 10^4^/µL)	37.0 ± 0.3	33.6 ± 0.2	0.40	35.6 ± 0.1	33.2 ± 0.1	0.22
Alb, mean ± SD (g/dL)	3.0 ± 0.2	3.3 ± 0.1	0.15	3.0 ± 0.1	3.4 ± 0.1	< 0.01
CRP, mean ± SD (mg/dL)	7.9 ± 1.7	6.4 ± 1.3	0.50	7.7 ± 0.8	5.4 ± 0.6	0.02
NLR, mean ± SD	2.8 ± 0.2	2.9 ± 0.2	0.85	2.8 ± 0.1	2.9 ± 0.2	0.50
Number of metastases, mean ± SD						
Lung	2.4 ± 0.6	2.3 ± 0.5	0.90	2.3 ± 0.3	2.2 ± 0.2	0.94
Bone	0.1 ± 0.1	1.1 ± 0.4	0.02	0.1 ± 0.0	1.2 ± 0.2	< 0.01
Liver	0.4 ± 0.3	0.1 ± 0.1	0.33	0.5 ± 0.1	0.2 ± 0.0	0.12
Brain	0.1 ± 0.1	0.1 ± 0.1	0.50	0.1 ± 0.0	0.0 ± 0.0	0.42

Longer OS; ≥ median OS, Shorter OS; < median OS, *IPTW* inverse probability of treatment weighting, *IMDC* the International Metastatic RCC Database Consortium, *CN* cytoreductive nephrectomy, *ECOG PS* ECOG performance status, *Hb* hemoglobin, *Alb* albumin, *CRP* C-reactive protein, *NLR* neutrophil-to-lymphocyte ratio, *SD* standard deviation

The original publication has been updated.

