# Supplementary material for: Significance of upfront cytoreductive nephrectomy stratified by IMDC risk for metastatic renal cell carcinoma in targeted therapy era – a multi-institutional retrospective study
Source: Int J Clin Oncol. 2022 Jan 1;27(3):563–73. doi: 10.1007/s10147-021-02091-8 (PMC8882566; doi:10.1007/s10147-021-02091-8)
Supplement: Supplementary file 1 — Supplementary file1 (DOCX 41 KB) [file 10147_2021_2091_MOESM1_ESM.docx]

**Supplementary Table 1. Variables included in propensity score model for treatment with upfront CN vs. non-upfront CN.**

| Age | |
| --- | --- |
| Sex | |
| Number of IMDC risk: | IMDC intermediate risk: 2  IMDC poor risk: ≥ 4 |
| ECOG PS | PS ≥ 1 |
| T stage | cT3b / 3c / 4 |
| N stage | cN1 / 2 |
| M stage | cM1 |
| Metastatic sites | Lung  Bone  Liver  Brain  Lymph node |

IMDC; the International Metastatic RCC Database Consortium, ECOG PS; ECOG performance status

**Supplementary Table 2. Drugs used as first line therapy in upfront CN group and non-upfront CN group.**

|  | Upfront CN | Non-upfront CN |
| --- | --- | --- |
| IMDC intermediate risk |  |  |
| Targeted therapy, n (%)  Sunitinib  Sorafenib  Axitinib  Pazopanib | 34 (50.8)  7 (10.5)  4 (6.0)  6 (9.0) | 22 (38.6)  10 (17.5)  19 (33.3)  2 (3.5) |
| Cytokine therapy, n (%)  IFN  IFN + IL2  Other | 8 (11.9)  2 (3.0)  6 (9.0) | 4 (7.0)  0 (0.0)  0 (0.0) |
| IMDC poor risk |  |  |
| Targeted therapy, n (%)  Sunitinib  Sorafenib  Axitinib  Pazopanib  Temsirolimus  Everolimus | 17 (50.0)  1 (2.9)  9 (26.5)  1 (2.9)  0 (0.0)  2 (5.9) | 40 (44.4)  6 (6.7)  34 (37.8)  0 (0.0)  8 (8.9)  0 (0.0) |
| Cytokine therapy, n (%)  IFN  Other | 2 (5.9)  2 (5.9) | 2 (2.2)  0 (0.0) |

**Supplementary Table 3. Comparing use of various types of systemic therapy in upfront CN group and non-upfront CN group.**

| Types of therapy | Unadjusted cohort | | | Adjusted cohort | | |
| --- | --- | --- | --- | --- | --- | --- |
|  | Upfront CN | Non-upfront CN | p-value | Upfront CN | Non-upfront CN | p-value |
| IMDC intermediate risk |  |  |  |  |  |  |
| Drug therapy, n  Cytokine / TT | 16 / 51 | 4 / 53 | 0.01 | 27 / 93 | 14 / 111 | 0.03 |
| Subsequent NIVO monotherapy, n (%) | 26 (37.1) | 9 (15.8) | < 0.01 | 42 (34.4) | 19 (15.4) | < 0.01 |
| Metastasectomy, n (%) | 21 (30.0) | 6 (10.5) | < 0.01 | 37 (29.2) | 18 (14.3) | < 0.01 |
| RT for bone metastases, n (%) | 19 (27.1) | 6 (10.5) | 0.02 | 26 (20.9) | 17 (13.4) | 0.09 |
| RT for brain metastases, n (%) | 9 (12.9) | 1 (1.8) | 0.02 | 16 (12.7) | 3 (2.1) | < 0.01 |
| IMDC poor risk |  |  |  |  |  |  |
| Drug therapy, n  Cytokine / TT | 4 / 30 | 2 / 88 | 0.04 | 16/104 | 2 / 123 | < 0.01 |
| Subsequent NIVO monotherapy, n (%) | 10 (27.0) | 11 (11.6) | 0.04 | 30 (20.9) | 15 (11.5) | 0.05 |
| Metastasectomy, n (%) | 2 (5.4) | 5 (5.3) | 1.00 | 6 (4.7) | 7 (5.2) | 1.00 |
| RT for bone metastases, n (%) | 7 (18.9) | 18 (19.0) | 1.00 | 29 (21.7) | 23 (17.3) | 0.53 |
| RT for brain metastases, n (%) | 3 (8.1) | 9 (9.5) | 1.00 | 12 (8.8) | 12 (8.8) | 0.83 |

CN; Cytoreductive nephrectomy, TT; targeted therapy, RT; radiation therapy

**Supplementary Table 4. IPTW-adjusted univariate and multivariate Cox regression analyses of systemic therapies predicting overall survival in IMDC intermediate risk group.**

| Covariates | | Univariate analysis | | | Multivariate analysis | | |
| --- | --- | --- | --- | --- | --- | --- | --- |
|  |  | HR | 95% CI | p-value | HR | 95% CI | p-value |
| Age | ≥ 75 vs 75 > | 1.45 | 0.94 – 2.25 | 0.09 | 1.96 | 1.12 – 3.42 | 0.02 |
| Sex | Men vs Female | 0.70 | 0.46 – 1.07 | 0.10 | 0.89 | 0.51 – 1.56 | 0.69 |
| Number of IMDC risk: | 2 risks vs 1 risk | 1.55 | 1.00 – 2.39 | 0.04 | 1.14 | 0.65 – 1.99 | 0.64 |
| ECOG PS | ≥ 1 vs 0 | 1.23 | 0.75 – 2.01 | 0.42 | 1.94 | 0.91 – 4.19 | 0.09 |
| T stage | ≥ cT3 vs cT2 ≥ | 2.38 | 1.45 – 3.89 | < 0.01 | 1.95 | 1.04 – 3.66 | 0.04 |
| N stage | cN1/2 vs cN0 | 1.97 | 1.23 – 3.14 | < 0.01 | 2.22 | 1.15 – 4.30 | 0.02 |
| M stage | cM1 vs cM0 | 1.12 | 0.58 – 2.17 | 0.74 | 0.69 | 0.29 – 1.65 | 0.41 |
| Metastatic sites  Lung  Bone  Liver  Brain  Lymph node | Yes vs No  Yes vs No  Yes vs No  Yes vs No  Yes vs No | 1.05  0.96  1.11  1.89  1.18 | 0.70 – 1.58  0.62 – 1.49  0.60 – 2.15  0.59 – 6.10  0.73 – 1.92 | 0.82  0.86  0.77  0.29  0.49 | 1.12  1.71  0.79  4.34  1.02 | 0.62 – 2.01  0.87 – 3.37  0.37 – 1.72  0.58 – 32.5  0.52 – 2.00 | 0.70  0.12  0.56  0.15  0.96 |
| CN | U-CN vs No-CN  U-CN vs D-CN  D-CN vs No-CN | 0.47  0.02  1.00 | 0.30 - 0.74  0.25 – 0.89  0.52 – 1.93 | < 0.01  0.02  0.99 | 0.44  0.72  0.61 | 0.24 – 0.81  0.31 – 1.65  0.24 – 1.54 | < 0.01  0.44  0.30 |
| First line drug therapy | Cy vs TT | 0.74 | 0.45 - 1.22 | 0.24 | 0.81 | 0.43 – 1.54 | 0.53 |
| Subsequent NIVO monotherapy | Yes vs No | 1.02 | 0.67 – 1.57 | 0.99 | 1.72 | 0.97 – 3.02 | 0.06 |
| Metastasectomy | Yes vs No | 0.81 | 0.48 – 1.36 | 0.43 | 0.92 | 0.49 – 1.74 | 0.80 |
| RT for bone metastases | Yes vs No | 0.97 | 0.57 – 1.66 | 0.92 | 1.04 | 0.52 – 2.08 | 0.91 |
| RT for brain metastases | Yes vs No | 0.60 | 0.25 – 1.47 | 0.27 | 0.54 | 0.16 – 1.75 | 0.30 |

U-CN; upfront cytoreductive nephrectomy, D-CN; deferred cytoreductive nephrectomy, No-CN; no cytoreductive nephrectomy, Cy; cytokine therapy, TT; targeted therapy, NIVO; nivolumab, RT; radiation therapy, N.D.; not detected
