# Supplementary material for: Significance of upfront cytoreductive nephrectomy stratified by IMDC risk for metastatic renal cell carcinoma in targeted therapy era – a multi-institutional retrospective study
Source: Int J Clin Oncol. 2022 Jan 1;27(3):563–73. doi: 10.1007/s10147-021-02091-8 (PMC8882566; doi:10.1007/s10147-021-02091-8)
Supplement: Supplementary file 2 — Supplementary file2 (DOCX 628 KB) [file 10147_2021_2091_MOESM2_ESM.docx]

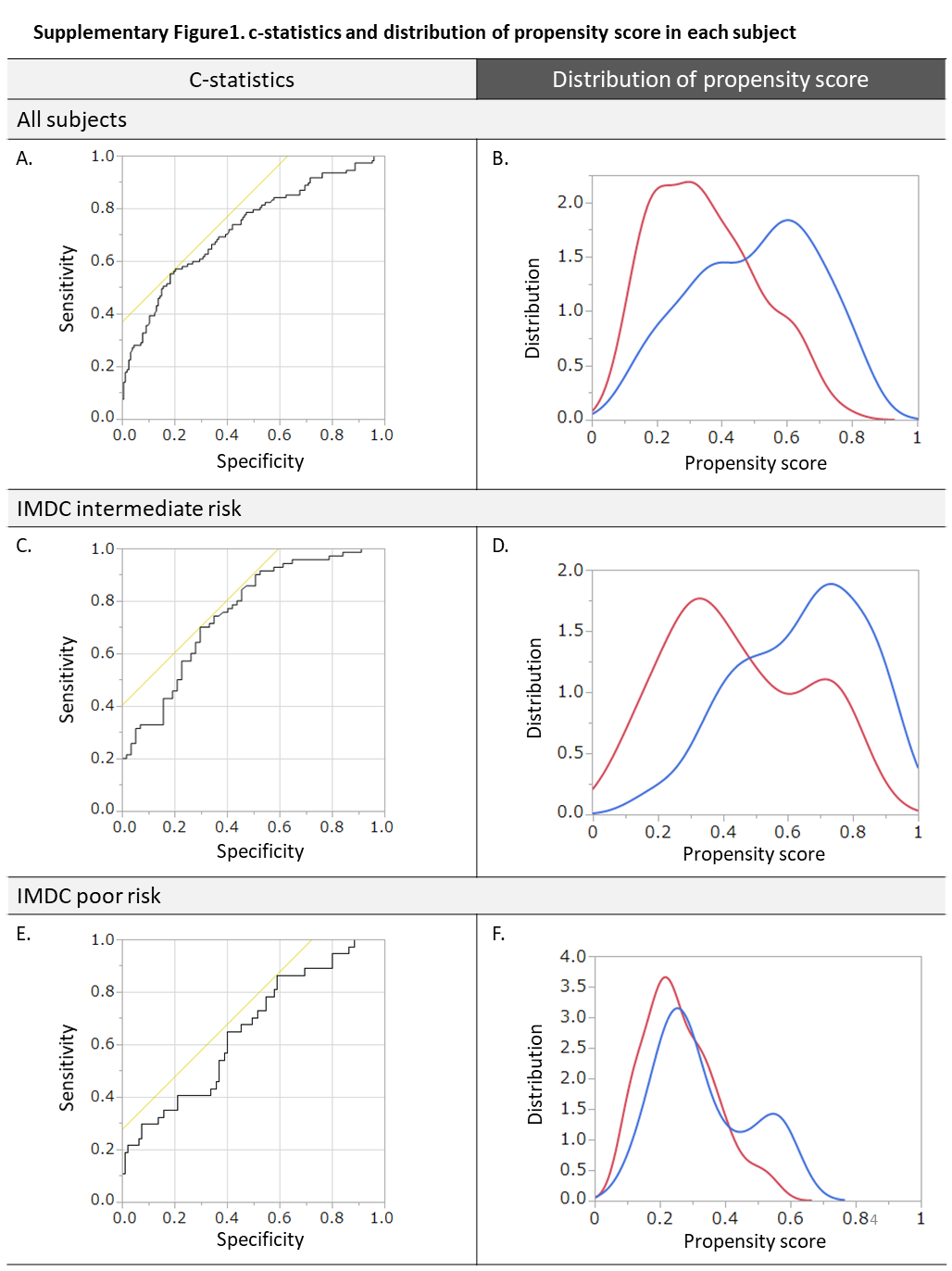


**Supplementary Figure 1. C-statistics and distribution of propensity scores.** Red curve represents non-upfront CN group, and blue curve represents upfront CN group.

All subjects: **A**. Receiver operating characteristic (ROC) curve generated by multiplex logistic analysis demonstrates an area under the curve of 0.72. **B**. Density distribution of propensity score shows moderate overlap of red line (upfront CN) and blue line (non-upfront CN). IMDC intermediate risk: **C.** ROC curve generated by multiplex logistic analysis demonstrates an area under the curve of 0.75. **D**. Density distribution of propensity score shows moderate overlap. IMDC low risk: **E.** ROC curve generated by multiplex logistic analysis demonstrates an area under the curve of 0.66. **F**. Density distribution of propensity score shows considerable overlap.


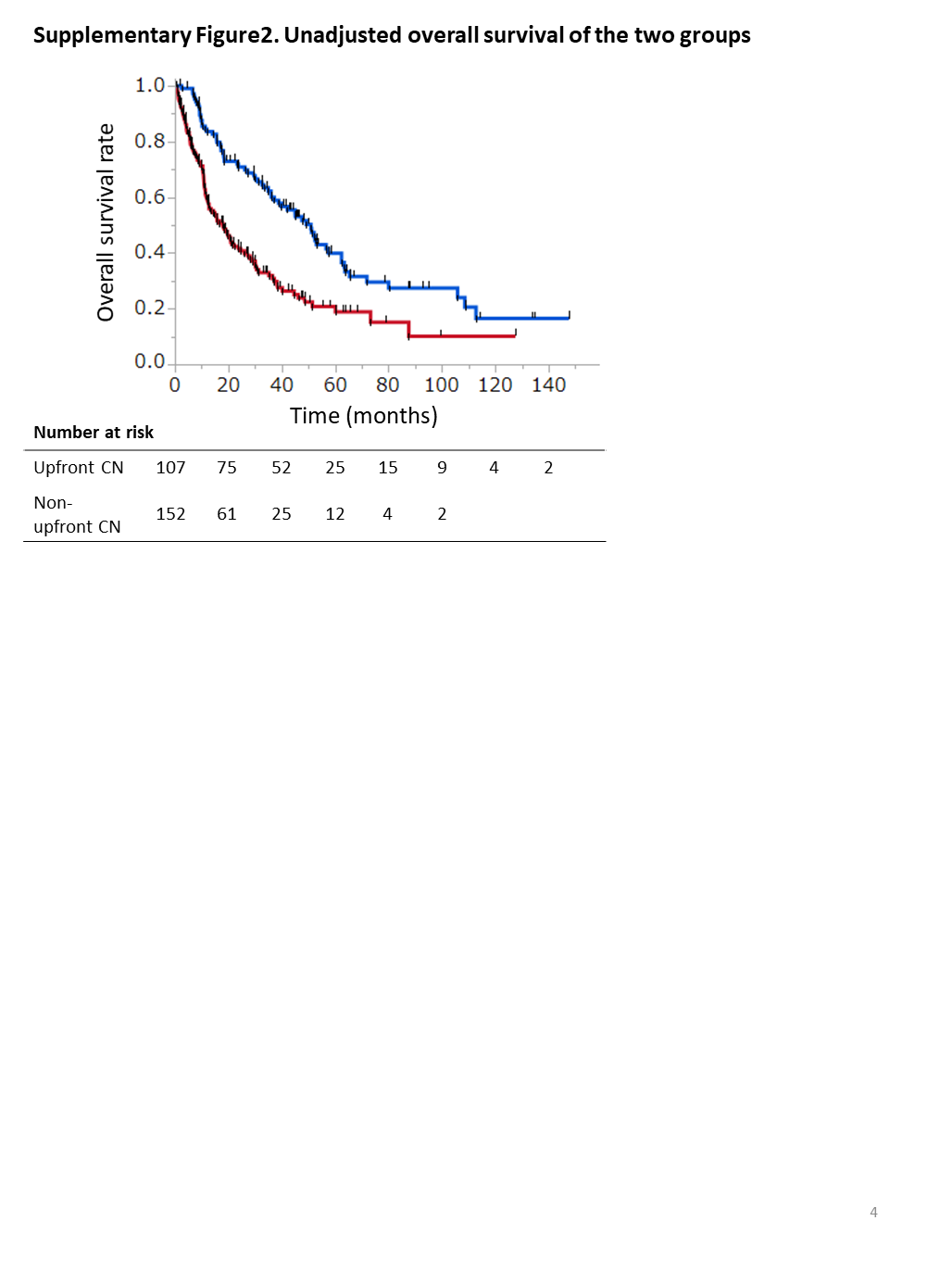


**Supplementary Figure 2. Unadjusted overall survival (OS) in all subjects.**

Red curve represents non-upfront CN group, and blue curve represents upfront CN group. Unadjusted OS [median (95% confidence interval)] is significantly longer in upfront CN group than in non-upfront CN group [50.9 (36.1–62.4) months versus 18.1 (12.4–23.8) months; p < 0.01].


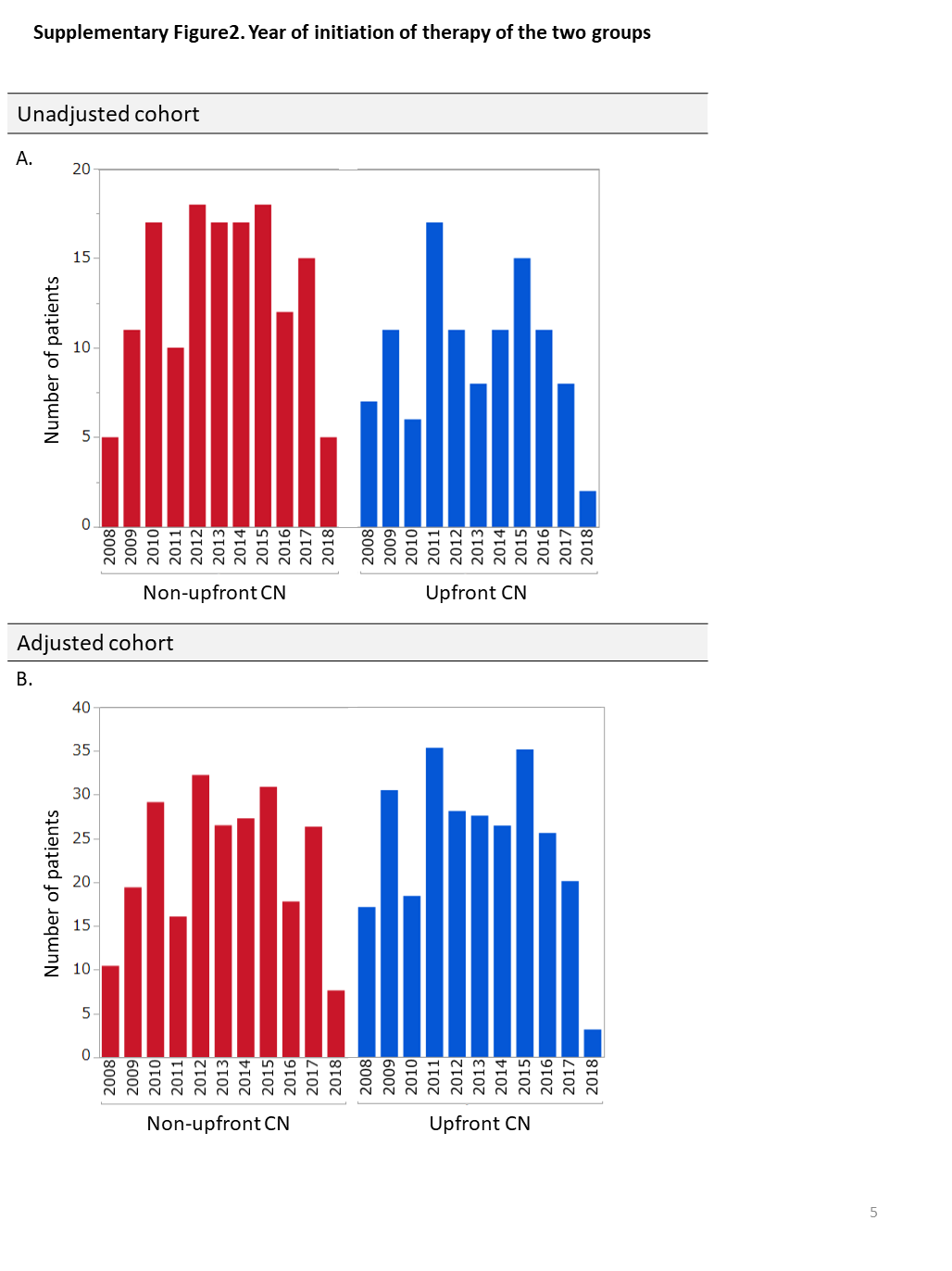


**Supplementary Figure 3. Year of initiation of first therapy in upfront CN and non-upfront CN groups.**

Red curve represents non-upfront CN group, and blue curve represents upfront CN group. **A**. Comparison of years of initiation of first therapy (year of surgery in upfront CN group and year of first-line drug therapy in non-upfront CN group) shows no significant difference between the two groups in non-adjusted cohort (p = 0.29). B. Comparison of years of initiation of first therapy showed no significant difference between the two groups in IPTW-adjusted cohort (p = 0.07). CN; cytoreductive nephrectomy**.**


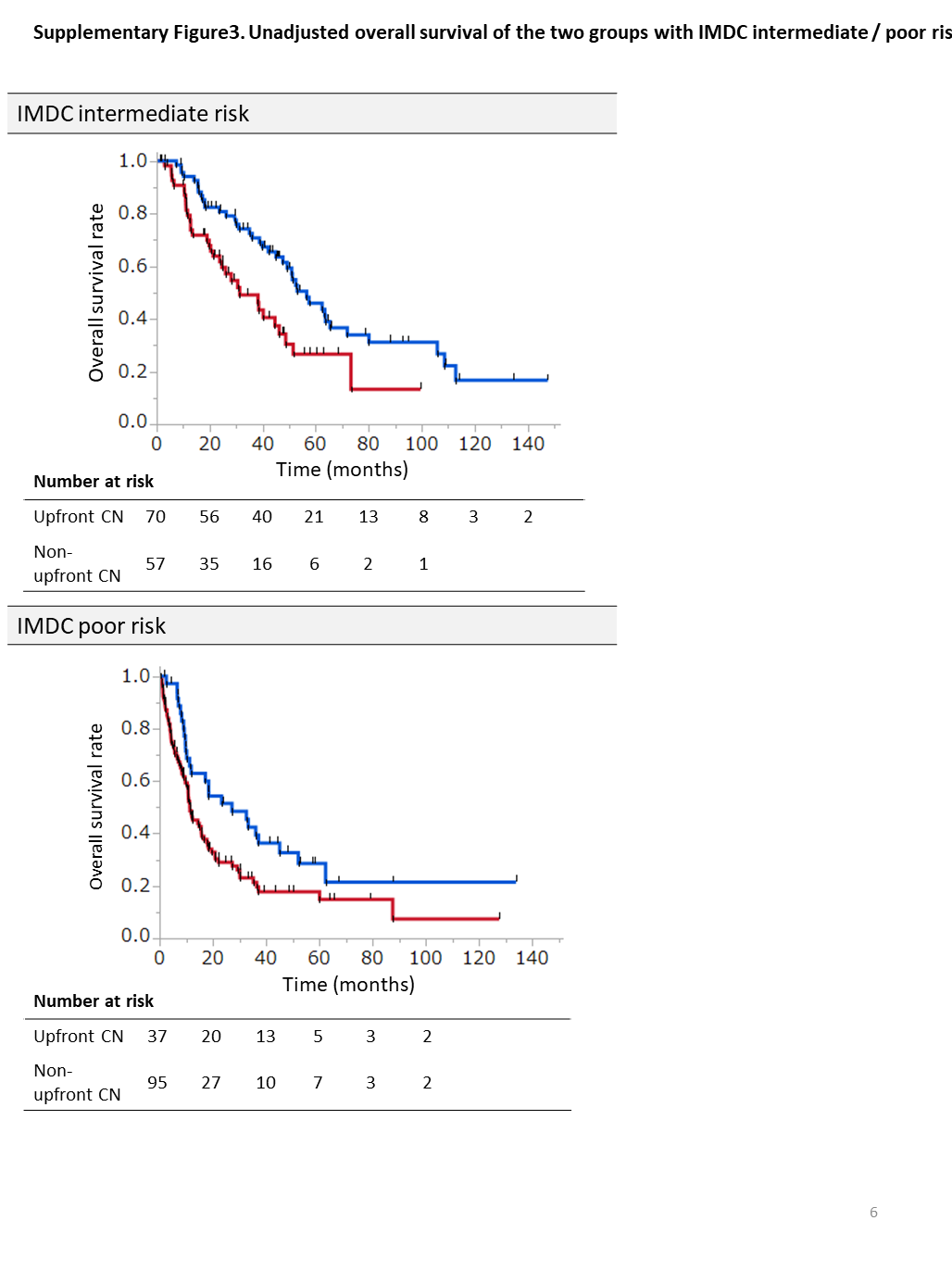


**Supplementary Figure 4. Unadjusted overall survival (OS) in patients with IMDC intermediate or poor risk.**

Red curve represents non-upfront CN group, and blue curve represents upfront CN group.

- **IMDC intermediate risk subgroup**

Unadjusted OS [median (95% confidence interval)] is significantly longer in upfront CN group than in non-upfront CN group [56.6 (47.6–65.4) months versus 31.3 (21.3–46.2) months; p < 0.01].

- **IMDC poor risk group**

Unadjusted OS [median (95% confidence interval)] is significantly longer in upfront CN group than in non-upfront CN group [27.2 (11.3 – 45.1) months versus 11.4 (9.6 – 15.8) months; p = 0.02].
